# Impact of Work Hours on the Quality of Life of Adult Employees With Irritable Bowel Syndrome in Saudi Arabia

**DOI:** 10.7759/cureus.31983

**Published:** 2022-11-28

**Authors:** Waleed M Alhuzaim, Abdullah M Alojayri, Fahed A Albednah, Faisal F Alshehri, Mohannad S Alomari, Meshal A Alyousef, Nahaa E Alsubaie

**Affiliations:** 1 College of Medicine, Imam Mohammad Ibn Saud Islamic University (IMSIU), Riyadh, SAU; 2 Department of Mathematics, AlKhurmah University College, Taif University, Taif, SAU

**Keywords:** saudi arabia, quality of life, employees, work productivity, work hours, irritable bowel syndrome

## Abstract

Introduction

Irritable bowel syndrome (IBS) is one of the most prevalent gastrointestinal disorders worldwide. There is still debate about the pathophysiology of IBS. Symptoms of IBS include abdominal pain and alternating bowel movements, but the severity differs among the patients, which affects their quality of life. Our main aim in this study is to find the impact of work hours on the quality of life of adult employees with irritable bowel syndrome in Saudi Arabia.

Methods

An analytical cross-sectional study was conducted using an online self-administered survey including employees over 18 years old in Saudi Arabia. The survey was designed in three different parts. The first part is demographics and personal information, The second concentrates on IBS using the Rome-IV criteria while the third part reviewed the participant's quality of life by utilizing the quality-of-life scale (QOLS).

Results

The total number of participants was 1800; most of the population were females (954; 53%) and there were 846 (47%) males. The study showed that 27.11% were diagnosed with IBS. Furthermore, the result revealed significant differences between working hours, with employees who work more than nine hours (33.7%) being more affected by IBS than others. Nevertheless, significant independent risk factors for IBS were QOLS (OR = 0.988; 95% CI (0.981, 0.995), p = .001), being an employee in free business (OR = 1.755; 95% CI (1.134, 2.714) p = .012), working between 6 and 9 hours (OR = 0.623; 95% CI (0.404, 0.961), p = .032).

Conclusion

The impact of work hours on adult employees with IBS in Saudi Arabia has been noticed; the results showed that the prevalence of IBS among females is higher; employees working more than nine hours with a medium to sedentary work nature are more vulnerable to developing IBS. We suggest that IBS patients should address their needs to their employers.

## Introduction

Irritable bowel syndrome (IBS) is one of the most common gastrointestinal chronic disorders. The controversy related to the pathophysiology of IBS usually influences the treatment plan. However, its effect on the patient's life is apparent although it varies from one patient to another depending on the severity of their clinical symptoms, which start with frequent intense abdominal pain, alternating bowel movements, and bloating [1,2]. IBS is diagnosed using the Rome-IV criteria, and according to a systematic review and meta-analysis, articles that utilized the Rome-III criteria had a greater prevalence than those that used the Rome-IV criteria [1,3]. IBS has long been known to have a negative impact on people's quality of life, similar to any other organic GI disease such as inflammatory bowel disease [4]. It is one of the most prevalent gut-brain association illnesses, affecting around one out of every 10 people globally [5,6].

The prevalence of IBS varies worldwide due to numerous factors. A systematic review and meta-analysis were conducted to identify the prevalence of IBS, and it was found that the estimated prevalence of IBS was (9.2%) using the Rome-III criteria and lower in the Rome-IV criteria (3.8%). Also, the study found that IBS is more prevalent among women than men (12.0% (95% CI 9.3-15.0) vs. 8.6% (6.3-11.2); odds ratio 1.46 (95% CI 1.33-1.59)) [1]. Nationally, the prevalence of IBS is 16.4% in Saudi Arabia, which is higher than the global prevalence [7]. In the central region of Saudi Arabia, a study aimed to identify the prevalence and risk factors of IBS. The prevalence was approximately (30.5%) [8]. Similarly, a study in Jizan, Saudi Arabia, estimated that the overall prevalence of IBS is 16%, which is higher than the worldwide range of IBS [9]. Likewise, a study aimed to determine IBS prevalence among board-certified physicians and surgeons at King Fahad Specialist Hospital, Dammam, Saudi Arabia. IBS prevalence was 16.3% overall, with no significant differences between gastroenterologists, surgeons, or pediatricians. Mixed IBS (IBS-M) was the most common type of IBS, affecting (45.4%) of individuals who had IBS. Diarrhea-predominant IBS (IBS-D) was the second-most common (32%), followed by constipation-predominant IBS (IBS-C) (20.6%) and unsubtyped IBS (IBS-U) (2.1%) [10]. Females outnumbered males in all of the previous national studies [7-10].

IBS is strongly associated with certain risk factors; the major is stress, including anxiety and depression. Stress levels highly influence the symptoms of IBS. Furthermore, there is a higher risk of IBS among females, but the specified cause of this association is ambiguous; positive family history is considered a risk factor but indecisive [1,2]. Besides, high anxiety and depression scale scores, working day shifts, and poor sleep quality were all strongly linked to IBS [11]. In addition, a cross-sectional study was conducted to explore the association of anxiety-depressive disorders with IBS in a family practice center. The study displayed that 62.9% of IBS patients suffered moderate to severe anxiety and depression, which was considered significantly higher than patients without IBS [12]. Another study found that IBS significantly impacts the patient's anxiety and despair levels and poor quality of life outcomes [13]. Still, data regarding the impact of IBS on daily activity and its association with psychological co-morbidities is insufficient, but they discovered that females, those who worked longer hours, and younger physicians were more likely to have IBS. Although, there was no link between having a particular specialty and IBS [10].

Some studies have aimed to identify the impact of IBS on work performance. In the United States of America (USA), patients diagnosed with IBS-D type have impacted their work productivity, daily activities, and missed workdays more than normal individuals. As a result, IBS patients will have reduced work productivity due to the symptoms. Nevertheless, the lost work hours have significantly impacted the indirect annual cost of around ($2486) more per employee per year in patients diagnosed with IBS-D, without mentioning the healthcare cost of managing this disorder. Although, there is no actual association between the different types of Rome-IV criteria and work productivity. IBS patients tend to be absent for approximately two days each month at work due to the symptoms of IBS [14]. In addition, it was estimated that (24.3%) of employees have gone absent due to IBS symptoms and have reported a considerable amount of work impairment associated with low quality of life, and more than half have reported presentism (productivity impairment while being at work) [15]. For this reason, the overall IBS-related work impairment is almost 81.8%, and though there are no specified predictors of absenteeism (work time missed due to illness), those with symptoms such as bloating and urgency compared with abdominal pain were less likely to report absenteeism [16].

Hence, IBS has a significant impact on work productivity, and it deteriorates work performance due to symptoms such as a feeling of generalized fatigue and gastrointestinal-related anxieties [15]. However, no studies were conducted to discover an association between IBS and work hours. Thus, our primary aim in this study is to find the impact of work hours on the quality of life among irritable bowel syndrome adult employees in Saudi Arabia. We also aimed to assess the possible risk factors for IBS in the study population and to estimate the prevalence of IBS among adult employees in Saudi Arabia.

## Materials and methods

Study design

This observational cross-sectional study was conducted in Saudi Arabia from April 2022 to June 2022. The study population included employees aged 18 years or more who presently live in one of the five regions in Saudi Arabia (central, western, southern, eastern, or northern). We calculated the sample size based on the Saudi Arabian population, which is approximately 35 million. So, the minimum sample size needed to achieve a precision of ±5% with a 95% confidence interval, resulting in 385 subjects. A convenience sampling technique was used for data collection; eventually, the data were collected using an online survey through data collectors who applied voluntarily for all five regions to collect data and distribute the questionnaire on social media and among their acquaintances.

Application of the questionnaire

The survey was designed into three different parts. The first part focuses on the demographic and personal information of participants, such as gender, age, marital status, occupation, and most importantly, job nature and the number of working hours. The second part concentrated on manifestations of irritable bowel syndrome; this part was done using a Rome-IV diagnostic questionnaire for adults. While the third part reviewed the participants' quality of life by utilizing the quality-of-life scale (QOLS) as a measurement tool.

The Rome Foundation released and designed the Rome-IV Diagnostic Questionnaire in 2016, and permission was provided by the foundation. The questionnaire was created to meet a requirement for putting the Rome-IV diagnostic criteria into the format of a question to simplify research and clinical screening. The IBS module of the questionnaire contains six questions and is utilized as a data-assembling tool. Participants who answer "Once a week" or a greater frequency for the first question, "30%" or greater for the second to the fourth question, and "Yes" for the fifth question are termed IBS positive. The last question divides who has IBS manifestations into IBS subtypes. The questionnaire tool has a sensitivity of 62.7% and a specificity of 97.1% in the case of IBS [3,17].

QOLS was founded by American psychologist John Flanagan in the mid-1970s. Then in 2003, it was adapted to concentrate its main scope on chronic illness populations by Burckhardt, Carol & Anderson, Kathryn. QOLS has 16 items that mainly concentrate on five basic fields of quality of life: material and physical well-being, relationships with other people, social-community and civic activities, personal development and fulfillment, and finally independence. The scores range from 16-112, with an average of 90 for healthy populations. The higher score suggests a better quality of life. The QOLS is a reliable score scale for chronic illnesses with a Cronbach's coefficient of α = 0.82 to 0.92 [18].

Ethical consideration

Institutional review board approval was obtained from the Medical Research Unit, College of Medicine, Al-Imam Mohammed ibn Saud Islamic University, Riyadh, Saudi Arabia, based on the Declaration of Helsinki. Participation in this study was completely voluntary; every participant was informed to give their consent and they were welcome to participate. The participants were not gaining any materialistic earnings due to their participation.

Statistical analysis

After the collection of data, the variables were revised and entered, and statistical analyses were conducted using Statistical Package for Social Sciences (SPSS) version 28.0 (IBM Corp, Armonk, NY). All data that are missing any variable were excluded from the study. Continuous variables were represented as mean ± standard deviation (SD). The frequency and percentage were used for categorical variables. Chi‑square was used to compare categorical variables. Univariate analysis was utilized to check the relation between IBS and each independent variable. A p-value of < 0.05 was considered statistically significant. Binary logistic regression was used to predict the likelihood of IBS using different factors like QOLS, job, residence, work nature, working hours, gender, and age. The analysis of variance (ANOVA) test was conducted to assess the effect of working hours on QOLS. Levene’s test was used to check the assumption of the homogeneity of variance.

## Results

**Figure 1:**
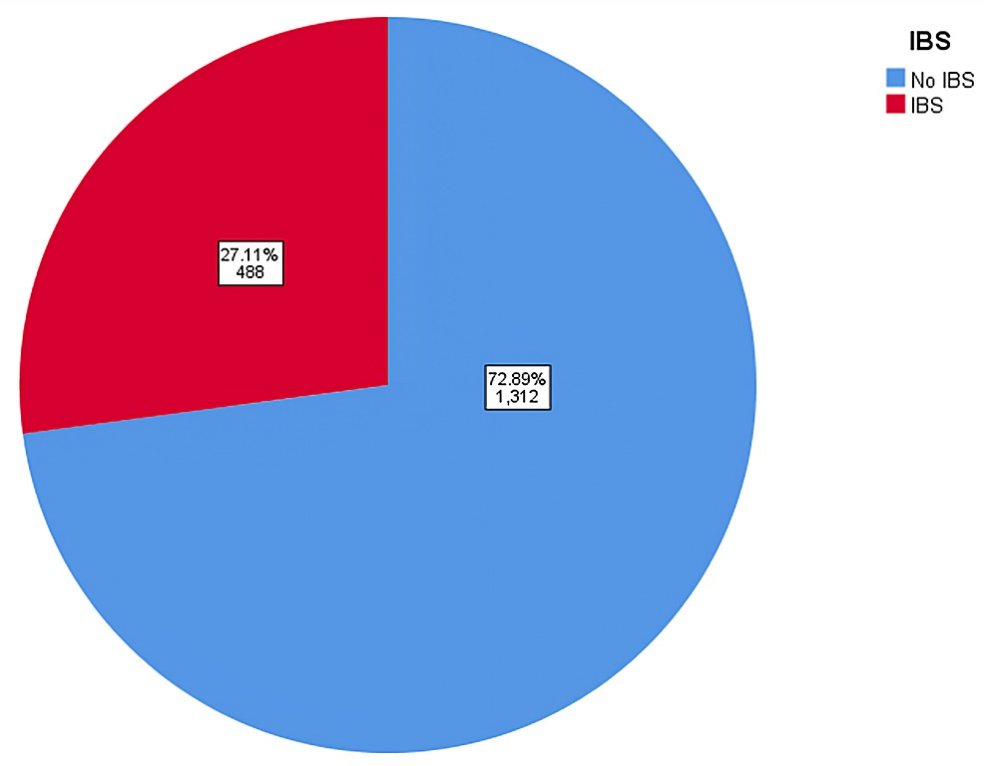
Prevalence of IBS IBS: Irritable Bowel Syndrome

**Figure 2:**
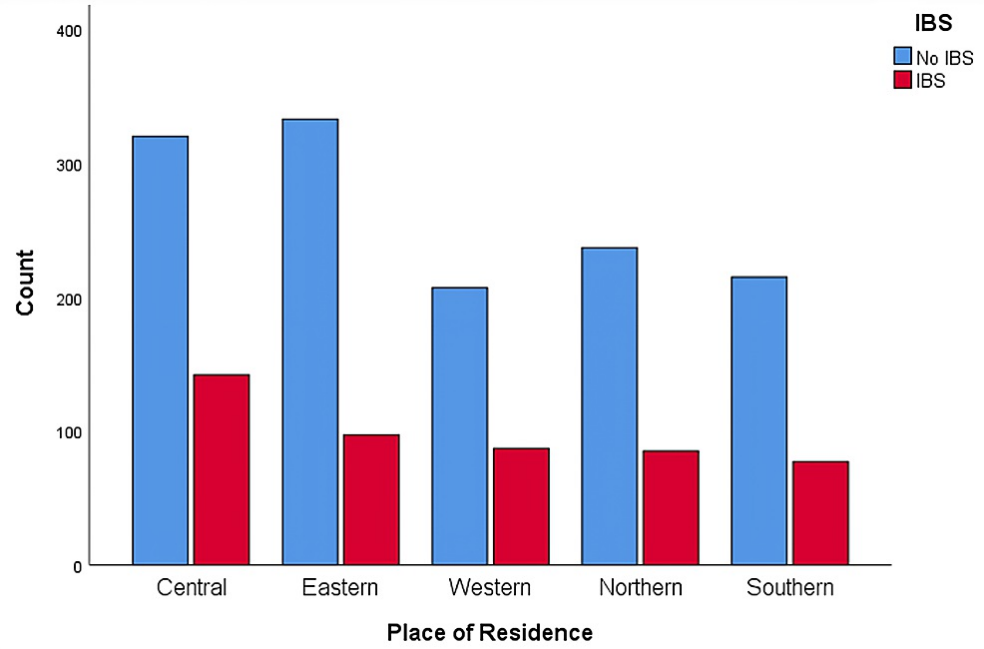
Prevalence of IBS in each geographical region of Saudi Arabia IBS: Irritable Bowel Syndrome

A total of 1800 subjects participated in the study in the present survey. As depicted in Table 1, in the study sample, the majority of participants were in the age group of 18-25 (44.6%), 846 (47%) were males, and 954 (53%) were females. As for nationality, 1719 (95.5%) were from Saudi Arabia. Regarding occupation, 690 (38.3%) were working in the governmental sector, 257 (14.3%) were in the private sector, and 763 (42.4%) were students. The nature of work varied between sedentary (714; 39.7%), medium active (881; 48.9%), and 205 (11.4%) active. It was found that 462 (25.7%) of the participants were from the central region of Saudi Arabia, 430 (23.9%) from the eastern region, 294 (16.3%) from the western region, 322 (17.9%) from the northern region, and 292 (16.2%) from the southern region (Table 1). The assessment of the Rome-IV diagnostic criteria revealed that the prevalence of IBS was 488 (27.1%) study population who met the criteria for an IBS diagnosis (Figure 1). Regionally, the prevalence of IBS in the central region is 142 (30.7%), followed by the western region (87; 29.6%), northern region (85), southern region (77; 26.4%), and the eastern region (97; 22.6%) (Figure 2). 

**Table 1:** Baseline characteristics of 1800 participants N: Number; IBS: Irritable Bowel Syndrome

Parameters		N	%
Gender	Male	846	47
	Female	954	53
Age	18-25 Years	802	44.6
	26-35 Years	430	23.9
	36-45 Years	333	18.5
	26-55 Years	201	11.2
	56-65 Years	29	1.6
	65+ Years	5	0.3
Saudi	Not Saudi	81	4.5
	Saudi	1719	95.5
Job	Government	690	38.3
	Private Sector	257	14.3
	Free Business	77	4.3
	Non_Profit	13	0.7
	Student	763	42.4
Work Nature	Sedentary	714	39.7
	Medium	881	48.9
	Active	205	11.4
Residence	Central Region	462	25.7
	Eastern Region	430	23.9
	Western Region	294	16.3
	Northern Region	322	17.9
	Southern Region	292	16.2
IBS	No IBS	1312	72.9
	IBS	488	27.1

**Figure 3:**
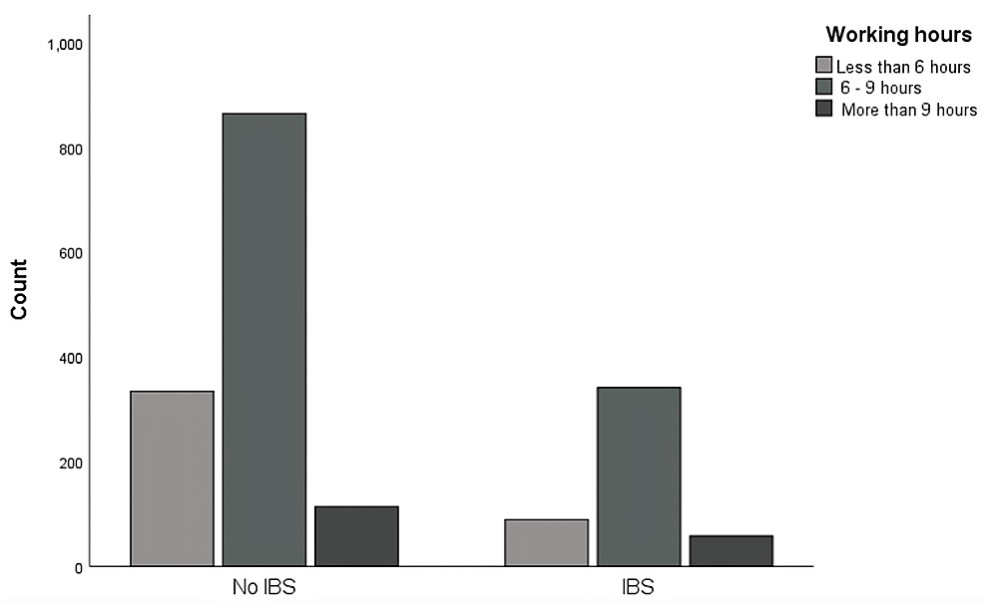
Clustered bar chart for IBS and working hours IBS: Irritable Bowel Syndrome

Regarding the associated risk factors with IBS symptoms, Table 2 represents a univariate analysis that displays that job type (χ2 = 15.17, p = .004) showed significant differences among different job types and working hours illustrated statistical significance (χ2 = 12.55, p = .002). The result demonstrated significant differences between working hours, with employees who work more than 9 hours (33.7%) being more affected by IBS than others who work 6-9 hours (28.3%) and less than 6 hours (21%). Although gender, age, nationality, and work nature did not show statistical significance for the association with IBS (p > 0.05). And Figure 3 displays the differences in working hours between IBS and non-IBS.

**Table 2:** Variables associated with IBS IBS: Irritable Bowel Syndrome

Characteristics		IBS	No IBS	χ2	p
Gender	Male	214	632.0	2.66	0.103
	Female	274	680.0		
Age	18-25 Years	209	593.0	7.93	0.160
	26-35 Years	136	294.0		
	36-45 Years	91	242.0		
	26-55 Years	44	157.0		
	56-65 Years	7	22.0		
	65+ Years	1	4.0		
Saudi	Not Saudi	21	60.0	0.06	0.806
	Saudi	467	1252.0		
Job	Government	181	509.0	15.17	0.004
	Private Sector	92	165.0		
	Free Business	22	55.0		
	Non-Profit	6	7.0		
	Student	187	576.0		
Work Nature	Sedentary	204	510.0	2.94	0.230
	Medium	223	658.0		
	Active	61	144.0		
Working Hours	Less than 6 Hours	89	334.0	12.55	0.002
	6-9 Hours	341	864.0		
	More than 9 Hours	58	114.0		

Binary logistic regression was conducted to ascertain the effects of QOLS, job, residence, work nature, working hours, gender, and age on participants' likelihood of IBS. The logistic regression model was statistically significant, χ2(22) = 51.28, p < .001. The model explained 4.6% of the variance in IBS and correctly classified 67.8% of cases. Table 3 summarizes the results of the regression analysis of possible risk factors for IBS in the study population. Significant independent risk factors for IBS were QOLS (OR = 0.988; 95% CI (0.981, 0.995), p = .001), being an employee in free business (OR = 1.755; 95% CI (1.134, 2.714) p = .012), working between 6 and 9 hours (OR = 0.623; 95% CI (0.404, 0.961), p = .032).

**Table 3:** Multiple regression analysis: risk factors for IBS among employees IBS: Irritable Bowel Syndrome

Risk factors	B	S.E.	p	OR	95% C.I.	
QOLS	-0.012	0.004	0.001	0.988	0.981	0.995
Job - Government	Ref					
Private Sector	0.256	0.214	0.232	1.292	0.849	1.967
Free Business	0.562	0.223	0.012	1.755	1.134	2.714
Non-Profit	0.265	0.318	0.404	1.304	0.699	2.432
Student	0.887	0.605	0.143	2.427	0.741	7.947
Residence - Central Region	Ref					
Eastern Region	0.027	0.179	0.882	1.027	0.723	1.459
Western Region	-0.289	0.191	0.130	0.749	0.515	1.088
Northern Region	0.155	0.202	0.444	1.168	0.785	1.736
Southern Region	-0.081	0.198	0.682	0.922	0.625	1.360
Work Nature - Sedentary	Ref					
Medium	-0.142	0.192	0.460	0.868	0.596	1.264
Active	-0.276	0.192	0.151	0.759	0.521	1.105
Working Hours: Less Than 6 Hours	Ref					
6-9 Hours	-0.474	0.221	0.032	0.623	0.404	0.961
More Than 9 Hours	-0.153	0.190	0.420	0.858	0.592	1.244
Gender - Male	-0.099	0.121	0.416	0.906	0.715	1.149
Age: 18-25 Years	Ref					
26-35 Years	1.025	1.141	0.369	2.787	0.298	26.076
36-45 Years	0.870	1.143	0.447	2.386	0.254	22.424
26-55 Years	0.768	1.149	0.504	2.155	0.227	20.487
56-65 Years	0.550	1.157	0.634	1.734	0.180	16.731
65+ Years	0.624	1.232	0.613	1.866	0.167	20.855

**Figure 4:**
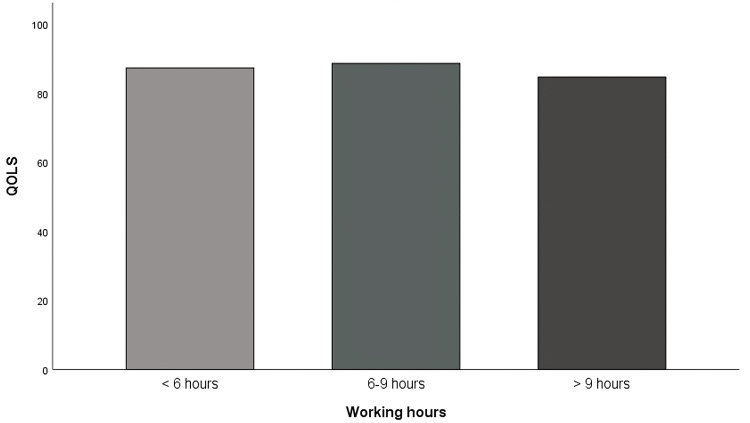
Bar graph comparing the difference in means of QOLS between working hours QOLS: Quality of Life Scale

The average score of IBS using QOLS is 85.7, the lowest score among IBS participants was 31, and the highest was 112 (Table 4). A one-way ANOVA was conducted to assess the effects of working hours on QOLS. The assumption of homogeneity of variance was checked using Levene’s test, and the result was not significant, F(2,1521) = 5.13, p = .006. This indicates that the variances are not equal. Hence, Brown-Forsythe will be reported. The result implied a significant difference in QOLS between the working hours' groups, F (2,422.06) = 3.86, p = .022. A follow-up test was conducted using Games-Howell for pairwise comparisons between the groups. The result also showed that there is a significant difference in QOLS between working hours for 6-9 hours (M = 88.70, SD = 15.41) and more than 9 hours (M = 84.75, SD = 18.49) with a mean difference of 3.95, p = .039, 95 % CI (0.16, 7.74) (Figure 4).

**Table 4:** QOLS scores with IBS QOLS: Quality of Life Scale; IBS: Irritable Bowel Syndrome

QOLS		N	Minimum	Maximum	Mean	SD
No IBS		1036	17	112	89.11	15.25
IBS		488	31	112	85.71	16.54

## Discussion

This article aimed to assess the impact of work hours on the quality of life of adult employees with IBS in Saudi Arabia. Our study has revealed that the prevalence of IBS among Saudi employees is 27.1% higher than the worldwide prevalence in a systematic review and meta-analysis conducted by Oka et al. [1]. Similarly, multiple studies conducted by Chang et al. in Asian countries and Hungin et al. across eight European countries showed a lower overall prevalence of IBS than our population [19,20]. Moreover, Bin Abdulrahman et al. conducted a study on a nationwide scale in the Kingdom of Saudi Arabia, where the results showed a lower IBS prevalence [7]. As the result implied, the highest prevalence of employees with IBS was in the central region of Saudi Arabia (30.7%). Equivalently, Aljammaz et al. conducted a study in the same region and revealed a similar prevalence of 30.5% [8]. The southern region had an IBS prevalence of (16%) in the study done by Arishi et al. in Jazan [9]. However, the result revealed a higher IBS prevalence among employees in the same area (26.4%). Additionally, Alharbi et al. performed a study in the northern region, where the overall prevalence of IBS was 11.8% [21]. Counter to the result, there was a higher IBS prevalence among employees in the same region (26.4%). This prevalence variance may result from using different data collection tools, socioeconomic differences between nations, or regional limitations. Furthermore, the sample size can be the reason for this discrepancy.

In our study, female participants were more prevalent than male participants, the same as in all the studies conducted in the central, northern, and southern regions of Saudi Arabia [8,9,21]. Internationally, a study across Europe presented a female predominance, as in Saudi Arabia [20]. However, the study done by Long et al. in South China reported no appreciable differences in the proportions of male and female participants [22]. These differences might be attributed to sex steroid hormones, menstrual cycle, and psychological status [19]. This discrepancy could be caused by the social status, psychological burden, and illness-related behaviors that differ between men and women in various populations.

This article revealed a statistical significance that showed a good quality of life could have protective measures from IBS exacerbation on employees diagnosed with IBS according to the odd ratio. Our results revealed that non-IBS employees have a higher mean quality of life score than IBS employees' mean scores of (89.1 and 85.7, respectively) on QOLS. A study disclosed that the prevalence of anxiety among IBS participants had double the percentage compared to non-IBS participants, decreased scores on the Short Form-8 (SF-8) for quality of life, and higher Hospital Anxiety and Depression Scale (HADS) scores for anxiety and depression [22]. Another study by Alruwaili et al., conducted in Al-Jouf, Kingdom of Saudi Arabia, among Saudi males, found that almost half of the participants reported that IBS had an emotional impact on them and limited their social and recreational activities [23]. IBS patients scored significantly lower than patients with chronic disorders such as gastroesophageal reflux disease (GERD), diabetes mellitus, and renal disease requiring dialysis on the Short Form-36 (SF-36) Health Survey, which assesses health-related quality of life [24]. A study by Choghakhori et al. to determine the sex-related differences in clinical symptoms and quality of life among IBS patients indicated that compared to males, women have more severe IBS symptoms and scores as compared to males lower on the IBS-QOLS [25]. Finally, a significant independent risk factor on the quality of life among IBS employees, such as being employed in a free business, demonstrates a better quality of life based on the odd ratio. This could be due to multiple convenient factors, such as flexibility of work hours, controlling the work environment, and fewer physical and mental stressors, which intensify the quality of life.

Our results have shown significant differences in the risk of IBS among employees working in Saudi Arabia, as employees who work more than 9 hours have the highest chance of getting affected by IBS (33.7%) while other employees who work 6-9 hours and less than 6 hours have less chance of getting affected by IBS (28.3% and 21% respectively). Eventually, increased work hours lead to poorer quality of life as our results uncover a lower mean of 84.75 among employees who work more than 9 hours and a mean of 88.7 among employees who work 6-9 hours. This is in line with our hypothesis that employees who work longer hours suffer more from IBS exacerbation compared to others who work shorter hours. As well as a study conducted by AlAmeel et al. on board-certified medical doctors in Saudi Arabia concluded that IBS was more prevalent in younger physicians who worked longer hours [10]. Furthermore, IBS employees who work more than six hours have a higher possibility of facing IBS manifestations than others who work less than six hours. This could lead to an impact on their work productivity in different aspects, as seen in a previously published article conducted by Frändemark et al. on IBS employees reported that the degrees of absenteeism and presenteeism (24.3% and 86.8%, respectively) are tightly associated with increasing severity of IBS symptoms that lead to overall work productivity loss both absenteeism and presenteeism. As well as the quality of life of IBS employees is inversely correlated with work productivity and activity impairment [15]. On top of that, a study on IBS patients with the IBS-D subtype by Buono et al. found that respondents with IBS-D missed significantly more work hours, expressed higher levels of presenteeism while at work, experienced more significant overall work productivity loss, and incurred higher indirect costs when compared with non-IBS placing a significant burden on patients and employers [14]. Based on our results, many variables contribute to the risk of having IBS, such as being female, within 18-25 of age, working in the governmental sector, having a sedentary to mildly active work nature, and finally working 6-9 hours a day. In Saudi society, this age group of individuals is within the career-determining period, so they continuously have physical and psychological stressors, thus ignoring their physical and mental health. These sensitizing events may cause the central nervous system to become hypersensitive, a condition that can be precipitated by either internal or external stimuli. This sensitivity triggers IBS symptoms, primarily manifested in the enteric nervous system [26].

There are several limitations in our study that must be considered. This article used a convenient sampling technique, which might lack generalizability. We recommend conducting the study using a more advanced type of sampling. Since the study is cross-sectional, there is a possibility of recall bias. Also, the 13 provincial classifications were not used in the study, and the 5 geographic regions were used instead. Stress and intensity levels vary between occupations, which may affect the severity of IBS symptoms or the vulnerability to IBS; our study did not consider this. We suggest that future studies focus on finding a connection between specific professions and IBS in Saudi Arabia. We did not investigate the association between IBS subtypes and work hours. Further studies are needed to determine the other casual effects that can lead to absenteeism and presenteeism other than IBS symptoms in the workplace. Finally, prevalence variances may be brought about using various methods of data collection, national socioeconomic variances, sample size, or regional restrictions.

## Conclusions

The impact of work hours on irritable bowel syndrome adult employees in Saudi Arabia has been assessed. As our hypothesis states, reducing work hours for IBS patients is recommended to improve their quality of life. The results showed that the prevalence of IBS among females is higher; participants aged 18-25 or who work more than 9 hours with a medium to sedentary lifestyle are more vulnerable to IBS than others. Finally, as informed in this article, IBS patients should have special consideration regarding their needs to improve their personal, financial, and social quality of life. We also suggest that the executives make work hours more flexible, control the work environment, and reduce physical and mental stressors on their employees suffering from IBS, and we recommend that IBS patients be encouraged to openly address their necessities to their employers.
